# The Scrapping Mechanism for the Corundum–Mullite Refractory Material in Rotary Kiln Incinerators

**DOI:** 10.3390/ma18030470

**Published:** 2025-01-21

**Authors:** Zhunqin Dong, Zhongbing Wang, Zongwen Zhao, Yuxia Song

**Affiliations:** 1 School of Metallurgy and Environment, Central South University, Changsha 410083, China; 2 Shandong Humon Smelting Co., Ltd., Yantai 264109, China; 3 School of Environment and Chemical Engineering, Nanchang Hangkong University, Nanchang 330063, China; 4 College of Environmental Science and Engineering, Central South University of Forestry and Technology, Changsha 410007, China; yuxiasong@csuft.edu.cn

**Keywords:** corundum–mullite refractory material, bridging oxygen, chemical corrosion, amorphous phase

## Abstract

Corundum–mullite refractory material is an important material in rotary kiln incinerators due to its excellent properties, e.g., high temperature stability and chemical resistance, etc. However, in the process of use, the complexity of the sintering process will inevitably produce a large amount of spent corundum–mullite refractory material. Therefore, it is important to study the failure mechanism of corundum–mullite refractory material to prolong its service life. In this manuscript, the scrapping mechanism for the corundum–mullite refractory material was studied by XRD, XPS, SEM-EDS, FTIR, etc. The results indicate that chemical corrosion caused by impurity elements, such as Fe, Ca, Mg, Ti, etc., is one of the important scrapping mechanisms. The corundum structure remains stable throughout the service life, while mullite exhibits the opposite phenomenon. The Al-O-Si bonds in the mullite structure are depolymerized by impurity elements to release free tetrahedral structures, including the [AlO_4_] tetrahedron and [SiO_4_] tetrahedron. In the intervention of iron, the free tetrahedra, including [AlO_4_], [FeO_4_], and [SiO_4_] can bond with each other by sharing bridging oxygen (BO), probably forming Fe-O(BO)-Si, Fe-O(BO)-Al, and Al-O(BO)-Si in an Al_2_O_3_-SiO_2_-Fe_2_O_3_-Me_x_O_y_ (Me = Ca, Mg, Ti, etc.)-based amorphous phase. These findings provide theoretical support for prolonging the service life of refractory materials in rotary kiln incinerators.

## 1. Introduction

With industrialization and urbanization, an increasing amount of goods are being produced to satisfy people’s needs. Meanwhile, byproducts, i.e., waste, including municipal waste, household waste, medical waste, and other hazardous waste, are inevitably being produced [1]. Without proper disposal, these wastes can pollute the environment and threaten human health. Currently, secure landfills and incineration are the two widely used methods for waste disposal worldwide [2]. Compared with secure landfills, incineration can effectively minimize waste mass and volume by 70% and 90%, respectively, and recover energy in the form of heat and electricity [3,4]. In China, the quantity of incinerated municipal solid waste increased from 46.3 million metric tons in 2013 to 101.8 million metric tons in 2018 [5]. Therefore, the incineration process has become an increasingly important technique for eliminating waste for environmental protection.

It is believed that the most important material in the incineration process is refractory brick. Refractories are solid materials that can withstand high temperatures and maintain their mechanical function for a required period of time under all circumstances, even when in contact with corrosive liquids or gases [6]. Based on refractory material interactions with water, refractory bricks can be classified as acidic (alumina-silicate materials, silica, etc.), basic (magnesia, doloma, and spinel), or neutral (chromia and alumina refractories) [7]. The main index of performance of incineration systems is the destruction rate of harmful substances, and the key parameters that affect the destruction rate of harmful substances in the incineration kiln mainly include the incineration temperature, retention time, material mixing degree, and air intake. The incineration temperature is one of the important bases for the structural design and material selection of the lining of the incinerator. For harmful substances in different industries, the incineration temperature of the kiln is different, but it is generally controlled between 850 °C and 1000 °C [8,9]. The length of the residence time directly affects the degree of incineration and determines the volume of the furnace body. The residence time of flue gas should be greater than 2 s to reduce the production of gas pollutants [8]. To decompose all harmful substances and combustion products, it is necessary to strengthen the full contact mixing of air and waste and air and smoke and to expand the contact area. The different silica–calcium–aluminum phases in the material may not only have a certain impact on the melting temperature but also be related to the performance of the refractory brick, thus affecting the service life of the refractory brick [10,11]. The total worldwide production of refractory material is approximately 35–40 million tons per year [12], with applications in industrial sectors including metallurgy and cement kilns [13,14]. The service lifetime of refractory bricks can vary significantly depending on their materials and application scenarios. Generally, the service lifetime of silicate refractory bricks is 1 to 5 years, that of alumina refractory bricks is 3 to 8 years, and the service life of silicon carbide refractory bricks is relatively longer, usually 5 to 10 years, which means that a large amount of spent refractory material is produced [15]. It is estimated that up to 28 million tons of spent refractories are generated every year [6]. In addition, spent refractories may result in potential environmental pollution due to the presence of hazardous substances, such as heavy metals. Therefore, extending the service lifetime of refractories is of high importance for reducing the quantity of spent refractory material and for environmental protection.

Fundamental research on the scrapping mechanism of refractory materials in practical use is needed for taking inhibitory measures to prolong the service life of refractory materials and reduce the production of spent refractory materials. According to a previous study [16], the scrapping mechanisms for refractory materials can be classified into two categories, i.e., mechanical spalling and chemical corrosion. The presence of spent refractory materials is mainly caused by chemical corrosion. The service environment, including the temperature, atmosphere, material properties, and reactivity between materials and refractories, is linked to the corrosion behavior of refractory materials [17]. Yao et al. [18] studied the microstructure and physical properties of a mullite brick under different thermal treatment temperatures and found that the temperature can affect the microstructure of the mullite matrix. With an increasing temperature, the compressive strength and modulus of rupture increase, which is beneficial for the washing resistance of slag/iron from the mullite brick. Jansson et al. [19] studied the corrosion mechanism of a MgO-C refractory in contact with CaO-Al_2_O_3_-SiO_2_-MgO slag and found that the dissolution of refractory material into the slag was followed by the penetration of pores and grain boundaries and the dispersion of the grains into the slag. Szczerba et al. [20] suggested that the main mechanism of refractory brick corrosion was the formation of new phases by chemical corrosion reactions between the phases of basic brick and the phases of cement clinker; however, they did not specify what new phase was generated. Muhammed et al. [21] reported that alkali erosion is the main cause of damage to aluminum refractory bricks. When chromites migrated from hot to cold faces, the toughness of the magnesite brick decreased, and the brick flaked off during furnace cooling. Xu et al. [22] studied the degradation mechanisms of magnesia-chromite refractory bricks used in an oxygen side-blown reducing furnace. The results indicate that the infiltration of PbO-containing fayalite slag under turbulent conditions was responsible for the significant dissolution of periclase grains. Moreover, the FeO and ZnO in the slag were mainly absorbed by the chromite grains, which resulted in a large amount of monticellite in the infiltrated zone. Liu et al. [23] studied the corrosion of high-chrome refractory materials by high-sodium slag and found that the interaction between the high-chrome refractory material and high-sodium slag could occur throughout the entire penetration path until the Fe and Mg were exhausted. In addition, the presence of Na changed the characteristics of the slag, promoting its penetration into the refractory. Wang et al. [24] studied the corrosion reaction between the Cr_2_O_3_-Al_2_O_3_-ZrO_2_ refractory and slag and found that the molten slag entered the brick through the gaps and generated a spinel layer along the edge of the gaps, destroying the dense structures of the refractory surface. However, these works have focused mainly on the static or dynamic corrosion behavior of refractory materials at the laboratory scale, and less attention has been paid to the actual corrosion behavior of refractory materials.

The present study focused on the scrapping mechanism of refractory material from rotary kilns, which are often used to incinerate hazardous waste in China. Corundum–mullite refractory material is widely used in rotary kilns due to its advantages of remarkable thermal shock resistance, high temperature resistance, chemical corrosion, etc. [25,26]. Therefore, selecting corundum–mullite refractory material as the study object has representative significance. The scrapping mechanism of the corundum–mullite refractory material was studied by X-ray diffraction (XRD), thermogravimetric (TG) analysis, X-ray photoelectron spectroscopy (XPS), Fourier transform infrared (FTIR) spectroscopy, scanning electron microscopy–energy dispersive spectrometry (SEM-EDS), etc. These findings can be helpful for analyzing the scrapping mechanism of refractory material from a microscopic perspective and can then be used to take targeted measures to prolong the service life of refractory materials.

## 2. Materials and Analytical Methods

### 2.1. Refractory Material

**Figure 1:**
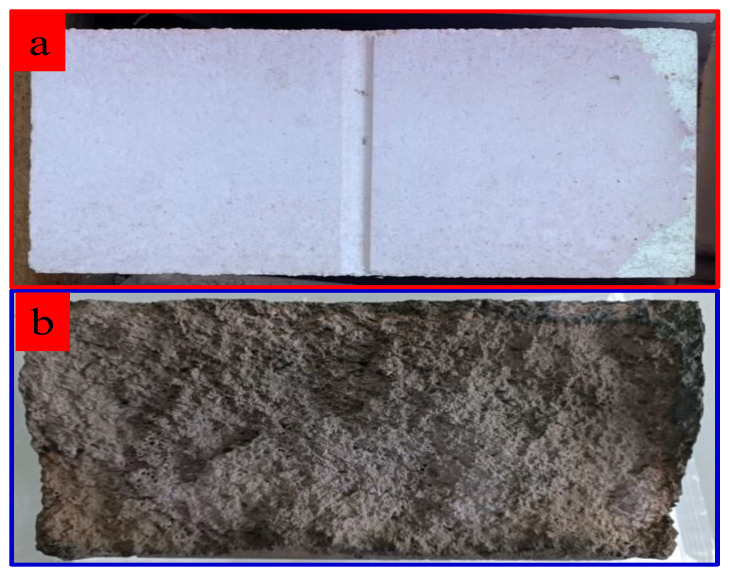
The appearance of new refractory material (**a**) and spent refractory material (**b**).

The refractory material was collected from an environmental protection enterprise in Zhuhai, China. The rotary kiln process was used by this company to dispose of industrial hazardous waste and medical waste. The appearances of the new and spent refractory materials are presented in Figure 1. The new refractory material has a regular rectangular shape with a dense surface, while the spent refractory material is ash black with an irregular shape, rough surface, uneven surface, and even a large deep pit, indicating that in addition to chemical erosion, there are other types of erosion, such as mechanical wear and tear, during the incineration process. Mechanical erosion destroys the compactness of the material surface and promotes chemical corrosion in the incineration process [27,28,29,30,31].

### 2.2. Analytical Methods

The chemical composition of the refractory material was measured using a Zetium PANAnalytical XRF spectrometer (Almelo, The Netherlands) (note: the compounds in the XRF measurement were present in the form of oxides or elements). The thermal stability of the new and spent refractory material were measured using thermogravimetric analysis. The specimens were placed on an Netzsch STA 449 C TGA apparatus (Selb, Germany) at temperatures ranging from room temperature to 1200 °C with the protection of argon flowing at a heating rate of 10 °C/min. The phase evolution and structural features of the refractory material were analyzed by XRD (Model D8 advance-A25), with 2θ values ranging from 5° to 80°. X-ray photoelectron spectroscopy (XPS) was conducted on a Thermo Scientific^TM^ K-Alpha^TM^+ spectrometer (Waltham, MA, USA) equipped with a monochromatic Al Kα X-ray source (1486.6 eV) operating at 100 W. All peaks were calibrated with a C1s peak binding energy of 284.8 eV for adventitious carbon. For Fe, Cu, and S, the spectra were deconvolved by subtracting the linear background and using a Gaussian (80%)–Lorentzian (20%) mixed function [32]. The micromorphology and elemental distribution were observed by the Hitachi S-3400 N instrument SEM-EDS (Tokyo, Japan). FTIR spectra were collected in the range of 400–2000 cm^−1^ using a BRUKER, VERTEX70 spectrometer (Karlsruhe, Germany) at a 4 cm^−1^ resolution using the KBr pellet technique.

## 3. Results and Discussion

### 3.1. Chemical Composition and Thermal Stability Analysis

#### 3.1.1. Chemical Composition Analysis

The chemical compositions of the new and spent refractory materials are presented in Table 1. The main chemical components of the new refractory material are Al_2_O_3_ (87.89%) and SiO_2_ (10.50%). The other chemical compositions are less than 2%. These impurities can be classified into alkaline impurities (Na_2_O, CaO, MgO, Fe_2_O_3_, K_2_O, etc.), acidic impurities (P_2_O_5_), and other impurities (TiO_2_, Cr_2_O_3_, etc.). The spent refractory material exhibits different chemical compositions. The Al_2_O_3_ content decreases remarkably to 65.41%. The SiO_2_ and Fe_2_O_3_ contents significantly increase to 19.08% and 7.44%, respectively. In addition, the impurities, including the species and the relative content of impurities, also increase in the spent refractory material. In the process of incineration, the corrosive melt or gas may react with the refractory material and destroy the density surface of the refractory material. Moreover, impurities are introduced into the refractory material.

#### 3.1.2. Thermal Stability Analysis

**Figure 2:**
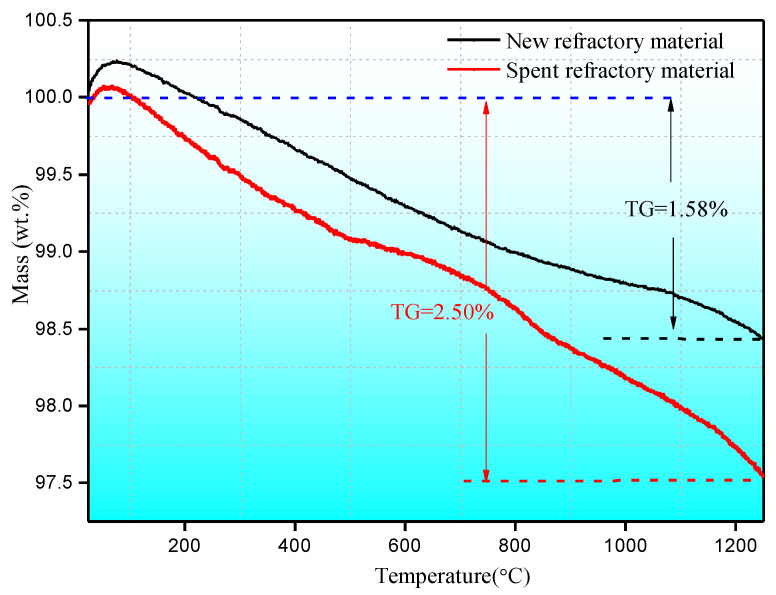
The TG curve for the refractory material.

TG has been used to study the thermal stability of the new and spent refractory materials, and the results are shown in Figure 2.

The refractory materials exhibit high thermal stability in the TG experiment corresponding to a temperature ranging from room temperature to 1200 °C. The whole weight loss values for the new and spent refractory materials are 1.58% and 2.5%, respectively. The weight loss is mainly derived from three parts, i.e., evaporable water, chemically bound water, and impurity volatilization. The weight loss caused by evaporable water and chemically bound water mostly occur below 600 °C. The remaining weight loss is possible due to the S- and Cl-containing impurity volatilization or metal salt decomposition. According to the chemical composition evolution in Table 1, the acidic impurities, such as S etc., and the metal impurities in the spent refractory material are higher than that in the new refractory material. The volatile property of S- and Cl-containing impurities is seen at high temperatures, resulting in more weight loss in the spent refractory material. In addition, the chemical decomposition/formation reactions, such as carbonates and other compounds formed in the alternating oxidation–reduction atmosphere. etc., is a possible cause for the higher weight loss in the spent refractory materials than that in the new refractory materials.

### 3.2. Phase and Chemical State Changes

#### 3.2.1. Phase Composition Changes

**Figure 3:**
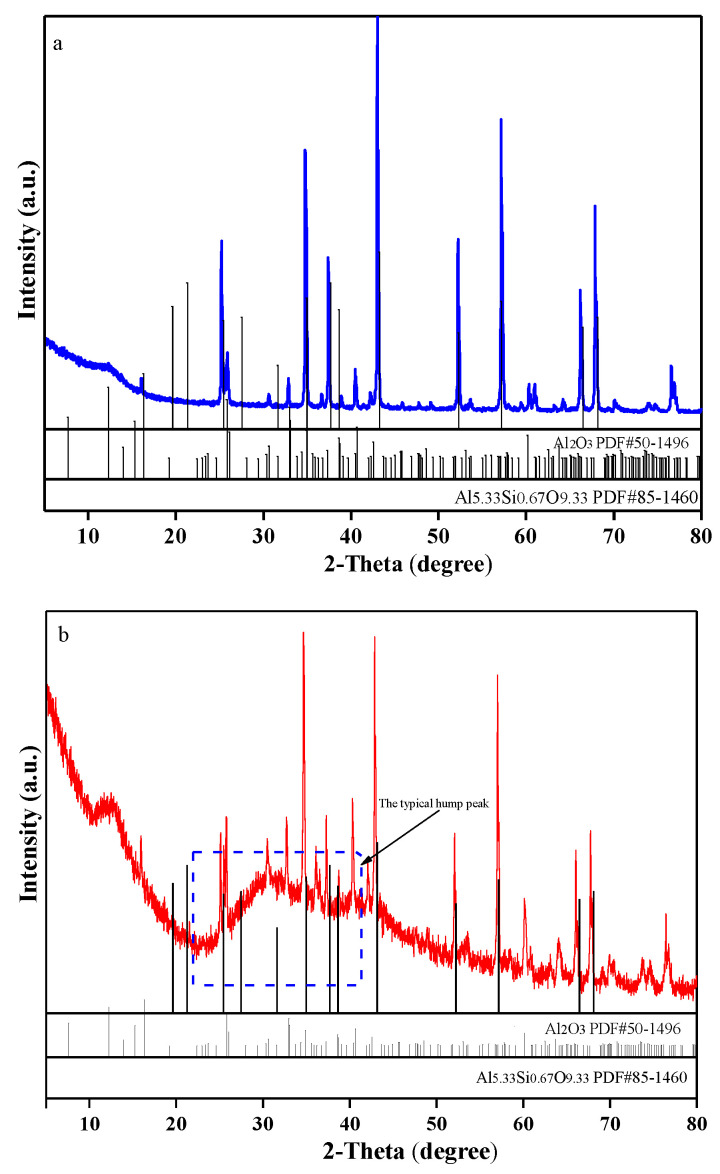
The XRD patterns for the new refractory material (**a**) and spent refractory material (**b**).

XRD was chosen to study the phase change in the new and refractory materials, and the results are shown in Figure 3.

The main phases in the new refractory material include Al_2_O_3_ (PDF#50-1496), and mullite corresponds to Al_5.33_Si_0.67_O_9.33_ (PDF#85-1460). Additionally, the Al_2_O_3_ and Al_5.33_Si_0.67_O_9.33_ phases are also observed in the spent refractory material, indicating that the phase change is not the immediate cause for the scrapping of the refractory material. However, the background noise of the XRD pattern increased, and a typical small broad hump peak (for 2θ = 20–40°) was observed in the spectrum of the spent refractory material, indicating the existence of an amorphous phase [33,34]. According to the chemical composition evolution in Table 1, the SiO_2_, Fe_2_O_3_, and Me_x_O_y_ (Me = K, Ca, Mg, etc.) contents are increased in the spent refractory material. The basic elements can reduce the amorphous phase formation temperature, inducing the formation of an amorphous phase. Additionally, the amorphous phase may weaken the stability of corundum–mullite refractory material.

#### 3.2.2. Surface Property Changes

**Figure 4:**
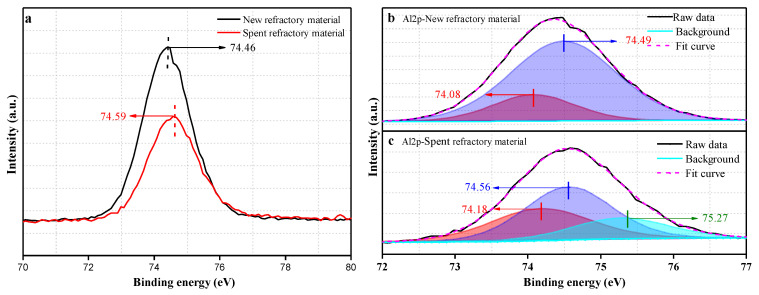
Al2p spectra for the new refractory material and spent refractory material (**a**) and the typical deconvoluted peaks (**b**,**c**).

**Figure 5:**
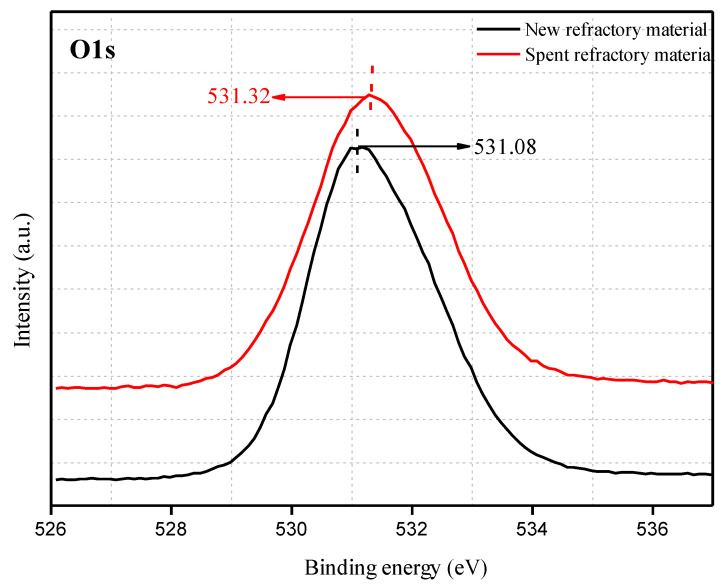
The O1s spectra for the new refractory material and spent refractory material.

**Figure 6:**
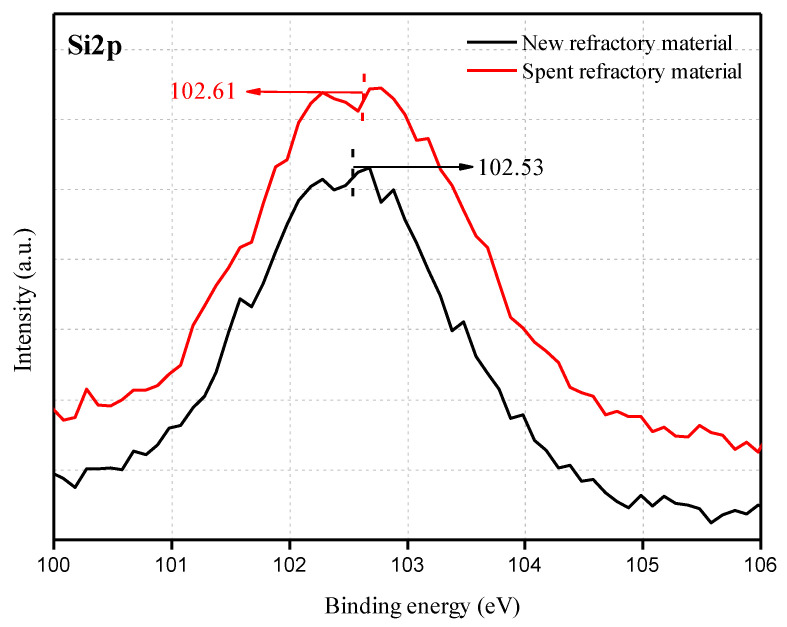
The Si2p spectra for the new refractory material and spent refractory material.

**Figure 7:**
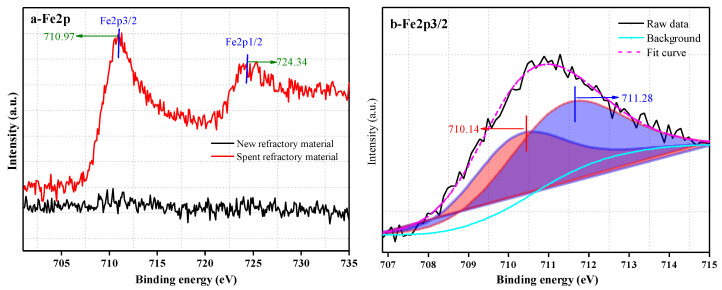
The Fe2p spectra for the new refractory material and spent refractory material (**a**) and the deconvoluted peak of Fe2p3/2 (**b**).

To further understand the properties of the amorphous phase in the spent refractory material, chemical bonding between the elements on the surface was studied by XPS, and the results are shown in Figure 4, Figure 5, Figure 6 and Figure 7.

The photoelectron spectrum of Al2p for the new and spent refractory materials is presented in Figure 4a. The Al2p peak of the new refractory material is located at 74.46 eV. The only Al-containing compounds present in the new refractory material are Al_2_O_3_ and mullite. Therefore, we assigned the binding energy of Al2p to the mixture of Al_2_O_3_ and mullite in the new refractory material. The Al2p peak of the spent refractory material significantly changed. First, the binding energy of Al2p shifted to a high peak area of 0.13 eV, changing from 74.46 eV to 74.59 eV, indicating that the chemical environment of the Al atoms has changed. In addition, the intensity of the Al2p peak significantly decreased. This indicates the loss of Al content in the spent refractory material, which was possibly caused by mechanical erosion during the incineration process. In addition, after deconvolution, two fitting peaks were located at approximately 74.08 eV and 74.19 eV. Previous studies [35,36,37] have shown that the binding energy of approximately 74.1 eV is due to the Al_2_O_3_ (corundum) compound, and the binding energy of approximately 74.2 eV is assigned to the aluminum atom in mullite. Therefore, we attributed the binding energies of the fitting peaks at approximately 74.08 eV and 74.19 eV to Al_2_O_3_ and mullite, respectively. The fitting peaks of the Al2p peak in the spectrum of the spent refractory material are at approximately 74.18 eV, 74.56 eV, and 75.27 eV. We believe it is reasonable to assign the binding energies of the fitting peaks at approximately 74.18 eV and 74.56 eV to Al_2_O_3_ (corundum) and mullite, respectively, due to the binding energy evolution in the new and spent refractory materials. The binding energy of the fitting peak near 75.27 eV was possibly due to other Al-containing compounds. XRD revealed that this compound is most likely an amorphous compound, including an Al-based matrix and amorphous aluminum oxide, for which the reported binding energies are ~75.0 eV [38] and ~74.4 eV [39,40], respectively.

The binding energy of O1s is presented in Figure 5. The binding energy of O1s is 531.08 eV for the new refractory material. O1s’s binding energy peak shifts to a high binding energy area with a maximum shift of approximately 0.24 eV in the spent refractory material, indicating a change in the oxygen chemical environment. For the new refractory material, three kinds of oxygen-containing units exist, namely, [AlO_6_] octahedra, [AlO_4_] tetrahedra, and [SiO_4_] tetrahedra [37,41,42]. In the spent refractory material, from the aspect of phase composition, a new formation of amorphous compounds may cause a shift in the O1s binding energy. Kim et al. [43] reported that the binding energies for bridging oxygen (the oxygen atom bonded to two glass-former cations), nonbridging oxygen (the oxygen atom bonded to a single glass-former cation), and free oxygen (the oxygen atom no bonded to glass-former cations) follow the order of bridging oxygen > nonbridging oxygen > free oxygen. In addition, the O1s binding energies for bridging oxygen, nonbridging oxygen, and free oxygen centers are approximately 532 eV, 531 eV, and 530 eV, respectively. Therefore, the greater shift in the binding energy of O1s in the spent refractory material is most likely due to the formation of bridging oxygen or nonbridging oxygen, which exists in the glass matrix. Therefore, the O1s binding energies reveal the formation of an amorphous phase in the spent refractory material.

The binding energy of Si2p is presented in Figure 6. The binding energies of the new and spent refractory materials are approximately 102.53 eV and 102.61 eV, respectively, with a maximum shift of approximately 0.08 eV. Due to the small binding energy shift, we believe that the Si2p binding energy change is probably due to a measurement error or decrease in the electron density around the Si atoms [44]. For the latter, the silicon source is derived from mullite in the refractory material. Therefore, the increase in Si2p is an index for the small-scale depolymerization of the mullite structure. The electron density of the silicon atom in a SiO_4_ tetrahedron can be significantly affected by the type of bonding for adjacent oxygen atoms. The only chemical bond present in the mullite structure is Si-O-Al. The [AlO_4_] tetrahedron and [SiO_4_] tetrahedron are glass formers, and the new probable formation of bridging oxygen in Si_te_-O-Al_te_ (te refers to tetrahedron) increases the binding energy of Si2p.

The Fe2p spectra of each sample are presented in Figure 7. Due to the low content of iron, the Fe2p peak is not present in the spectrum of the new refractory material. The Fe2p in the spectrum of the spent refractory material shows obvious Fe2p peaks, indicating a high content of iron. In addition, two satellite peaks attributed to Fe2p3/2 and Fe2p1/2 can be observed in the spectrum of the spent refractory material. The peak positions of Fe 2p3/2 and Fe 2p1/2 in our study are 710.97 eV and 724.34 eV, which are close to the values of 710.6 eV and 724.1 eV in the literature [45] and indicate the coexistence of Fe^3+^ and Fe^2+^ in the spent refractory material. Therefore, the deconvolution of Fe2p3/2 is shown in Figure 7b.

XRD did not detect any Fe-containing crystalline compounds, although the content of iron oxide in the spent refractory material was greater than 7%. Moreover, the strong background noise and the hump peak in the XRD indicate the existence of an amorphous phase. These phenomena prove that iron-containing compounds most likely exist in amorphous form. According to Figure 7b, the fitting peaks at approximately 710.14 eV and 711.28 eV correspond to Fe^2+^ and Fe^3+^. Due to the complex material composition, operating parameters, etc., the iron-containing material cannot be fully oxidized, resulting in the coexistence of Fe^2+^ and Fe^3+^ in the refractory material. Snow et al. [46] found that FeO has a powerful fluxing ability towards the A1_2_O_3_-SiO_2_ system, i.e., Fe^2+^ can partly depolymerize the mullite structure, releasing the [AlO_4_] tetrahedron and [SiO_4_] tetrahedron. In addition, Fe^3+^, Al^3+^, and Si^4+^ are glass formers [47,48] and can form bridging oxygens between each other, such as Fe-O(BO)-Si, Fe-O(BO)-Al, and Al-O(BO)-Si, resulting in the increased binding energies of O1s, Al2p, and Si2p.

### 3.3. Changes in Micromorphology and Elemental Distribution

**Figure 8:**
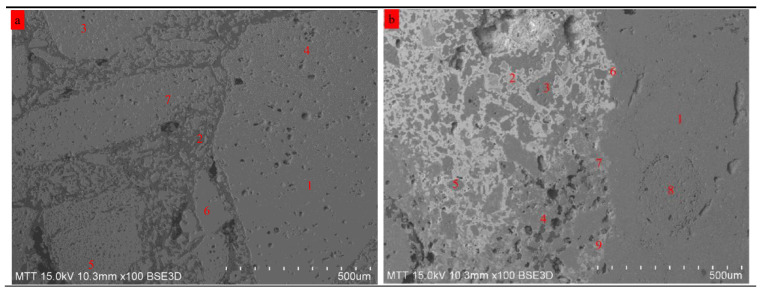
SEM images of the new refractory material (**a**) and spent refractory material (**b**).

The micromorphology and elemental distribution of the specimens are shown in Figure 8 and Table 2.

The polished new refractory material exhibits a homogeneous surface, with similar colors exhibited in Figure 8a. The EDS analysis, a semiquantitative test tool, was chosen to identify the compositions and phases in different regions. According to the EDS data, points 1, 4, and 5 are rich in Al, Si, and O and are therefore possibly assigned to mullite; points 3, 4, and 7 are rich in Al and O, the main phase of which is due to corundum; and point 2 is rich in C, which is mainly due to the resin originating from the specimen preparation process. In addition, this polished area exhibits little impurity elements, such as K, Ca, etc.

However, the surface of the spent refractory material shows marked differences in Figure 8b. First, the surface shows two shades, namely, bright and gray areas. To some degree, it is an index for the depolymerization of corundum–mullite materials. In addition, the elemental composition, i.e., the impurity element of this specimen, is more complex. Fe, Ca, Na, K, Mg, and Ti are found in the specimen. In particular, these elements, to some degree, can lower the formation temperature of the amorphous phase in the corundum–mullite refractory material. In other words, the depolymerization of the corundum–mullite structure is related to impurity element erosion. Similarly, the EDS data can also be classified into two parts. For the first part, the areas, including point 3 and point 8, are corundum-rich areas. The least amount of impurity elements (including only small amounts of Fe and Ca) are found in this part, indicating that the corundum structure is stable and difficult to erode during incineration. Thus, it possibly illustrates that the scrapping mechanism for the corundum–mullite refractory material is not caused by the corrosion of the corundum-rich phase. For the second part, the areas containing point 1, point 2, point 4, point 5, point 6, point 7, and point 9 are due to mullite. The element distribution characteristics prove that Fe, Mg, and Ti are the main impurity elements in the mullite-rich area, indicating that these three impurity elements have the ability to erode mullite. One guess is that the scrapping mechanism for the corundum–mullite refractory material is caused by the corrosion of the mullite-rich phase. In addition, based on the XRD result that proves the existence of the amorphous phase and the EDS results, it is reasonable to speculate that the amorphous phase is made up of Al_2_O_3_-SiO_2_-Fe_2_O_3_-Me_x_O_y_ (Me = Ca, Mg, Ti, etc.).

### 3.4. Microstructure Evolution

**Figure 9:**
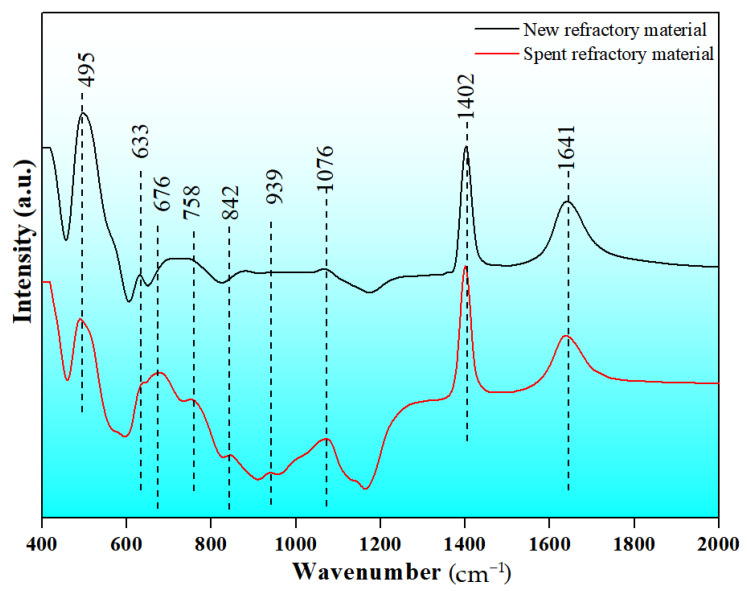
FTIR spectra for the new refractory material and spent refractory material.

FTIR was chosen to study the microstructure evolution of the new and spent refractory materials, and the FTIR spectra for the samples in the range of 400–2000 cm^−1^ are shown in Figure 9.

There are three obvious absorption peaks (495 cm^−1^, 1402 cm^−1^, and 1641 cm^−1^) and three weak absorption peaks (633 cm^−1^, 676 cm^−1^, and 758 cm^−1^) in the spectrum of the new refractory material. Additionally, the band at approximately 495 cm^−1^ is possibly due to the Si-O-Al rocking band [49,50], and the band near 633 cm^−1^ is a possible characteristic peak for the Al_2_O_3_·SiO_2_ structure [51]. The absorption bands at 676 cm^−1^ and 758 cm^−1^ are possibly due to the stretching vibration of Al-O bonds in the [AlO_4_] tetrahedron [52,53]. The bands located at 1402 cm^−1^ and 1641 cm^−1^ and exhibiting similar peak patterns and intensities in the spectra of the new and spent refractory materials can be attributed to the bending vibrations of O-H [54], molecular water, or hydroxyl-related bonds [55], which may be caused by the adsorption of water.

However, the FTIR peaks are specifically changed in the spectrum of the spent refractory material. First, the intensity of the peak, including the bands at 495 cm^−1^ and 633 cm^−1^, decreased. The Si-O-Al rocking band and Al_2_O_3_·SiO_2_ structure are the most likely characteristics of mullite in refractory materials. This change in the FTIR spectra indicates that the partial depolymerization of the mullite structure is due to the breaking of the Si-O-Al bond. Additionally, the intensities of several peaks, including those at 676 cm^−1^ and 758 cm^−1^, increase. This indicates the relative content of the [AlO_4_] tetrahedron in the spent refractory material. The Fe-, Mg-, and Ti-containing compounds can depolymerize the mullite structure and release the [AlO_4_] tetrahedron. Hence, the free [AlO_4_] tetrahedron can bond with the [SiO_4_] tetrahedron and [FeO_4_] tetrahedron by sharing the bridging oxygen. Finally, new bands at 842 cm^−1^, 939 cm^−1^, and 1076 cm^−1^ appeared. The band near 842 cm^−1^ may be ascribed to Si-O-Al linkages [51]. The new band at 939 cm^−1^ is possibly due to the overlapping vibrations of Si-O-Fe [56,57] and Si-O-Al [58]. The new band at approximately 1076 cm^−1^ can be attributed to the anti-symmetric stretching vibrations of the bridging Si-O-Si bonds within the [SiO_4_] tetrahedra [59]. The [FeO_4_] tetrahedron, [AlO_4_] tetrahedron, and [SiO_4_] tetrahedron are glass formers, and these free tetrahedron structures can form chemical bonds by bridging oxygen. Thus, new FTIR peaks at approximately 939 cm^−1^ and 1076 cm^−1^ are observed in the spectrum of the spent refractory material.

### 3.5. Hypothesis Failure Mechanism

**Figure 10:**
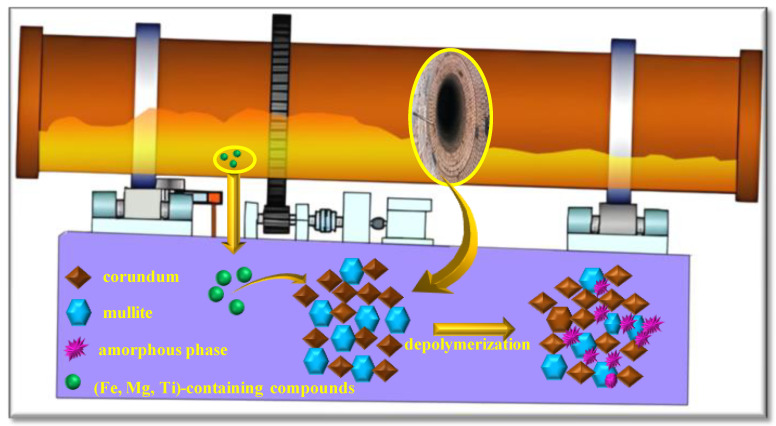
Hypothesis failure mechanism for refractory material.

In fact, the failure mechanism of refractory material is very complicated, and its failure process is related to many factors. Based on the research, a possible failure mechanism is proposed in this manuscript. The corundum has high chemical stability and is free of chemical depolymerization. While the mullite structure can be depolymerized by impurity elements in the refractory materials. The breaking of the Si-O-Al bond in mullite can release free tetrahedra, including [AlO_4_] tetrahedra and [SiO_4_] tetrahedra. With the intervention of glass formers, i.e., [FeO_4_], these tetrahedrons, including [AlO_4_], [FeO_4_], and [SiO_4_], can bond with each other by sharing bridging oxygen and form Fe-O(BO)-Si, Fe-O(BO)-Al, and Al-O(BO)-Si in the amorphous phase. The newly generated structure weakens the refractory materials’ stability and deteriorates the original mullite structure, inducing its failure (Figure 10).

## 4. Conclusions

To provide a deep understanding of the scrapping mechanism for the corundum–mullite refractory materials in rotary kiln incinerators, a comparative study was conducted. The XRD, XPS, SEM-EDS, and FTIR results for the new and spent corundum–mullite refractory materials were compared. The results indicate that chemical corrosion is one of the main causes of refractory material failure. The corundum structure remains stable throughout the service life. The partial depolymerization of mullite by impurity elements, such as Fe, Ca, Mg, Ti, etc., is an immediate cause for refractory material failure. Some Al-O-Si bonds in the mullite structure are depolymerized by impurity elements, releasing the free tetrahedra [AlO_4_] and [SiO_4_]. These free tetrahedra [AlO_4_], [FeO_4_], and [SiO_4_] can bond with each other by sharing bridging oxygen and form Fe-O(BO)-Si, Fe-O(BO)-Al, and Al-O(BO)-Si in the amorphous phase.

## Figures and Tables

**Table 1:** The chemical content of the new and spent refractory materials measured by XRF (wt.%).

Composition	New Refractory Material	Spent Refractory Material
Al_2_O_3_	87.89	65.41
SiO_2_	10.50	19.08
Fe_2_O_3_	0.72	7.44
Na_2_O	0.40	0.32
CaO	0.15	0.75
K_2_O	0.12	0.50
MgO	0.025	0.21
SO_3_	0.009	0.96
P_2_O_5_	0.01	1.24
TiO_2_	0.09	3.09
Cr_2_O_3_	0.01	0.05
MnO	0.04	0.06
Ga_2_O_3_	0.01	0.01
ZrO_2_	0.01	0.08
Co_3_O_4_		0.01
ZnO		0.24
SrO		0.04
CuO		0.05
NiO		0.02
PbO		0.01
CeO_2_		0.20
BaO		0.03
Cl		0.07

**Table 2:** Associated EDS data on the new refractory material and spent refractory material.

Elements	O	Al	Si	Fe	Ca	Na	K	Mg	P	Ti	C
New Refractory Material:											
Point 1	35.0	48.6	16.4								
Point 2	19.1	1.1	0.4								79.5
Point 3	32.2	67.8	0								
Point 4	36.0	47.1	16.9								
Point5	32.2	66.0	1.7								
Point 6	34.2	50.1	15.8								
Point 7	32.5	67.4	0.1								
Spent Refractory Material:											
Point 1	31.6	49.6	14.5	1.2						3.2	
Point 2	17.8	0.4	0.2	44.2				1.0		36.5	
Point 3	30.9	62.6	3.3	1.3	1.9						
Point 4	23.7	6.4	6.8	32.1	0.7			0.8	3.6	25.8	
Point5	31.9	45.2	12.7	2.0			1.0			7.2	
Point 6	31.8	40.8	13.6	5.7				0.6		7.5	
Point 7	30.4	31.9	29.8	2.8		1.0		0.4		3.7	
Point 8	25.3	73.0	0.3	1.5							
Point 9	31.1	26.0	27.3	1.0	8.9		1.4			4.3	

## Data Availability

The original contributions presented in this study are included in the article. Further inquiries can be directed to the corresponding authors.
